# Home-based initiatives for acute management of COVID-19 patients needing oxygen: differences across The Netherlands

**DOI:** 10.1186/s12913-023-10191-6

**Published:** 2023-11-15

**Authors:** Josi A. Boeijen, Alma C. van de Pol, Rick T. van Uum, Karin Smit, Abeer Ahmad, Eric van Rijswijk, Marjan J. van Apeldoorn, Eric van Thiel, Netty de Graaf, R. Michiel Menkveld, Martijn R. Mantingh, Silke Geertman, Nicolette Couzijn, Leon van Groenendael, Henk Schers, Jettie Bont, Tobias N. Bonten, Frans H. Rutten, Dorien L. M. Zwart

**Affiliations:** 1 Department of General Practice & Nursing Science, Julius Center for Health Sciences and Primary Care, University Medical Center Utrecht, Utrecht University, Heidelberglaan 100, Utrecht, 3584 CX The Netherlands; 2 Department of General Practice, Amsterdam University Medical Center, Location AMC, Meibergdreef 9, Amsterdam, 1105 AZ The Netherlands; 3 Primary Care Network Jeroen Bosch Huisartsen, Nieuwe Linie 231-232, Vught, 5264PJ The Netherlands; 4 Department of Internal Medicine, Jeroen Bosch Hospital, Postbus 90153, ’s-Hertogenbosch, 5200 ME The Netherlands; 5 Department of Pulmonology, Albert Schweitzer Hospital, Albert Schweitzerplaats 25, Dordrecht, 3318 AT The Netherlands; 6 Wilhelmina Hospital Assen, Europaweg-Zuid 1, Postbus 30001, Assen, 9400 RA The Netherlands; 7 Regional Organization for General Practice Drenthe, Dokter Drenthe, Stationsstraat 44, Assen, 9401 KX The Netherlands; 8 Department of Primary and Community Care, Radboud University Medical Center, Geert Grooteplein 21, Nijmegen, 6525 EZ The Netherlands; 9 Public Health & Primary Care, Leiden University Medical Center, Albinusdreef 2, Leiden, 2300 RC The Netherlands

**Keywords:** COVID-19, Oxygen therapy, Telemedecine, Home-based care, Hospital at home, Collaborative care

## Abstract

**Objective:**

During the COVID-19 pandemic new collaborative-care initiatives were developed for treating and monitoring COVID-19 patients with oxygen at home. Aim was to provide a structured overview focused on differences and similarities of initiatives of acute home-based management in the Netherlands.

**Methods:**

Initiatives were eligible for evaluation if (i) COVID-19 patients received oxygen treatment at home; (ii) patients received structured remote monitoring; (iii) it was not an ‘early hospital discharge’ program; (iv) at least one patient was included. Protocols were screened, and additional information was obtained from involved physicians. Design choices were categorised into: eligible patient group, organization medical care, remote monitoring, nursing care, and devices used.

**Results:**

Nine initiatives were screened for eligibility; five were included. Three initiatives included low-risk patients and two were designed specifically for frail patients. Emergency department (ED) visit for an initial diagnostic work-up and evaluation was mandatory in three initiatives before starting home management. Medical responsibility was either assigned to the general practitioner or hospital specialist, most often pulmonologist or internist. Pulse-oximetry was used in all initiatives, with additional monitoring of heart rate and respiratory rate in three initiatives. Remote monitoring staff’s qualification and authority varied, and organization and logistics were covered by persons with various backgrounds. All initiatives offered remote monitoring via an application, two also offered a paper diary option.

**Conclusions:**

We observed differences in the organization of interprofessional collaboration for acute home management of hypoxemic COVID-19 patients. All initiatives used pulse-oximetry and an app for remote monitoring. Our overview may be of help to healthcare providers and organizations to set up and implement similar acute home management initiatives for critical episodes of COVID-19 (or other acute disorders) that would otherwise require hospital care.

**Supplementary Information:**

The online version contains supplementary material available at 10.1186/s12913-023-10191-6.

## What is already known on this topic?

- Early discharge of COVID-19 patients is widely implemented in the Netherlands. In early discharge programs patients are sent home with oxygen after showing clinical improvement at the hospital ward.
- Acute home-based management of hypoxemic COVID-19 patients (i.e. without preceding admission to a ward other than the emergency department) is new in Dutch healthcare.
- A structured overview of initiatives for acute home-based management of hypoxemic COVID-19 patients is lacking.

## What this study adds

- We describe five acute home-based management initiatives in the Netherlands, all providing pulse-oximetry monitoring and an app for transferring measurements for remote review of values.
- Three initiatives included low-risk patients first evaluated at the emergency department, and two initiatives aimed at including frail patients who preferred not to be hospitalised.
- This article details different approaches to organize collaboration between professionals.

## Introduction

“Home management programs”, also called “hospital at home”, are initiatives that enable patients to receive treatments at home that would normally be provided in hospitals, in combination with home monitoring of vital parameters. These programs provide an opportunity to allow patients to recover in their home environment, alleviate pressure on healthcare systems, and potentially reduce costs [1–5]. Home management programs for pulmonary infections, heart failure exacerbations, and chronic obstructive pulmonary disease (COPD) or asthma exacerbations have been evaluated before the COVID-19 pandemic with promising results [6–8]. Home management programs for COVID-19 patients are expected to result in similar benefits [9, 10]. Almost all home management initiatives for COVID-19 patients have been “early discharge programs”, continuing treatment with oxygen at home after a patient has shown clinical improvement at the hospital ward first [11, 12]. Acute home-based management programs for COVID-19 patients – without initial hospitalisation – are a newer phenomenon, with the potential to avoid hospitalisation altogether.

The various acute home-based management programs for COVID-19 patients have in common that they require remote monitoring of patients’ vital signs and wellbeing (oxygen saturation, heart rate, severity of shortness of breath on a visual analogue scale, and sometimes breathing rate and blood pressure) and coordination of collaborative care organized around the patient staying at home. There are multiple approaches to organizing this collaboration, but an overview is currently lacking. We therefore aim to (i) present and analyse differences and similarities between Dutch “acute home-based management programs” (i.e. excluding “early discharge programs”), and (ii) provide insights in designing and implementing these new initiatives.

## Methods

### Initiative selection, inclusion criteria

Organization of acute home-based management programs can be initiated in primary care, emergency departments or in the hospital. In the Netherlands, the general practitioners (GPs) are central figures, as they have a gatekeeping function: referrals are required for both hospital and specialist care. Most GPs work independently or in a self-employed partnership and are regionally organized; organization of care may therefore vary across regions [13].

We identified initiatives by conducting a survey within the the Dutch General Practice Research Consortium. This consortium has representation from seven academic medical centers, as well as the Dutch College of General Practitioners and the Dutch Institute for Health Services Research [14]. We inquired whether any home management programs were implemented in the region. Subsequently, individual health institutions were approached. The inclusion criteria for eligible acute home-based management programs were:

- Patients were treated at home with oxygen and were monitored remotely.
- Patients were not admitted to the hospital prior to entering the acute home management program (except for a short admission lasting no longer than 24 hours).
- The time of onset of hypoxemia and the start of home management was less than 24 hours.
- Initiatives included at least one patient accordingly.

### Data collection

Relevant documentation was retrieved and evaluated. In case of uncertainties, healthcare centres’ staff members involved in the development of initiatives were contacted to obtain additional information. Similarities and differences were evaluated in five themes:

(i) Eligibility of patients
(ii) Organization of acute home treatment
(iii) Organization of remote monitoring
(iv) Organization of the nursing care at home
(v) Devices used.

All data was extracted in duplicate by two of our research team members (S.G., N.C., R.T.U. and/or J.A.B.), discrepancies were resolved by discussion with a third research team member.

## Results

### Eligible initiatives

Nine acute hom-based management initiatives were identified. Five were deemed eligible according to the predetermined inclusion criteria. The motivation for excluding the other four initiatives can be found in Supplementary Table 1. The five initiatives that were included were the following: the Jeroen Bosch Hospital Den Bosch (JB) the Wilhelmina Hospital Assen (WH), the Albert Schweitzer hospital Dordrecht (AS), and two initiatives from the University medical Center Utrecht (UT-1 and UT-2). In JB, WH, and AS, home-based management was organized within routine clinical practice, whereas in Utrecht it was initiated as a medical research project. Protocols from JB, WH, AS, and UT-1 and UT-2 were unpublished and obtained through personal communication. Temporal context on implementation and continuation of the initiatives can be found in Supplementary Table 2.

### Eligibility of patients

#### Clinical condition

All nine initiatives defined criteria to consider a patient eligible for home management with oxygen (Table 1). In JB, a patient was eligible if peripheral capillary oxygen saturation (SpO2) measured 93% or higher with a maximum of four litres of additional oxygen per minute. In WH, eligibility criteria were SpO2 90% or higher, respiratory rate of 24 breaths per minute or lower, and absence of signs of exhaustion. In AS, the eligibility was determined based on an arterial blood gas measurement (ABG) (patient was not eligible if the ABG was indicative of respiratory failure: PCO2 > 6.65 kPa, decreased PO2 and pH < 7.35). In both UT-1 and UT-2 patients were eligible in case of hypoxia (SpO2 93% or lower) or in case of laboured breathing (respiratory rate > 24 per minute), and without the need for intensive care according to the physician in charge.

**Table 1:** Eligibility criteria for the acute home-based management initiatives

	JB	WH	AS	UT-1^a^	UT-2^a^
Age in years	All^b^	All	≥ 18 years	≥ 18 years or < 80	≥ 18
Legally incompetent person^c^	Yes	Yes	No	No	No
Comorbidities					
Obesity, BMI in kg/m^2^	≤ 30	All	All	≤ 35	All
Heart failure	No severe comorbidities that required hospitalisation	Yes	Yes	No	Yes
Diabetes mellitus		Yes	Yes	No	Yes
Liver failure		Yes	Yes	No	Yes
COPD		Yes	Yes	No	Yes
Immunocompromised	No	Yes	Yes	No	Yes
Caregiver at home strictly required^d^	Yes	No	Yes	Yes	Yes
Digital proficiency required	Yes	Yes	Yes	No	No

*Abbreviations: BMI* Body mass index, *COPD* Chronic obstructive pulmonary disease

^a^UT-1 = non-frail patients who would otherwise have been treated in hospital, UT-2 = frail patient wanting to remain at home

^b^This information was obtained through interviewing staff

^c^Severe cognitive deficits e.g. in case of dementia or severe psychiatric condition

^d^Informal caregiver e.g. partner, family member or roommate

#### Patient characteristics and comorbidities

AS, WH, and UT-2 had no restrictions on comorbidities, whereas JB and UT-1 excluded patients with certain severe comorbidities such as obesity with Body Mass Index (BMI) ≥ 30 kg/m^2^ or severe COPD. WH and UT-2 explicitly aimed to manage frail patients in the home setting, whereas UT-1 and JB tailored the home intervention to patients for which a rather uncomplicated disease course was expected.

#### Caregivers

A partner, family member or other caregiver attending to the patient was required in all initiatives. These caregivers had to have proficiency in the Dutch language. AS, UT-1, UT-2 and JB specifically required that the caregiver lived with the patient for the duration of the home management.

### Organization of acute home treatment

#### ED evaluation and medical responsibility during home-based treatment

In three out of five initiatives (JB, AS and UT-1) patients were clinically evaluated at the ED prior to starting the home initiative (Table 2). In WH, patients were not evaluated at the ED if they needed fewer than three litres of oxygen treatment. If they needed more, ED evaluation was recommended. UT-2 did not require ED evaluation because these patients explicitly did not wish to visit the ED and hospital. The responsible physician during the home intervention differed across initiatives. GPs were responsible in JB, WH and UT-2, whereas hospital specialists were responsible in AS (pulmonologist) and UT-1 (either pulmonologist or internist).

**Table 2:** Medical care within the home management initiatives

	JB	WH	AS	UT-1^a^	UT-2^a^
ED evaluation	Yes	No. Only if > 3 L/min O_2_ was required	Yes	Yes	No
Medically responsible physician	GP	GP	Pulmonologist	Pulmonologist or internist	GP
Maximum O_2_ treatment (L/min)	4	3	3	5	5
Medical guidelines content^b^					
Corticosteroids	Yes				
Thrombosis prophylaxis	Yes				
Blood glucose check	Yes	Optional	Yes	Yes	Yes
Antibiotics	No	Optional	No	Optional	Optional
Person who gave instructions to the patient	ED nurse and case manager	GP^c^	Monitoring team	Researcher	Researcher

*Abbreviations: ED* Emergency department, *GP* General practitioner

^a^UT-1 = non-frail patients who would otherwise have been treated in hospital, UT-2 = frail patient wanting to remain at home

^b^For medical care details see Supplementary Table 3

^c^In everyday practice, this burdened the GPs too much, which hampered inclusion of patients. Therefore, an instruction team with (former) healthcare professionals was formed to instruct patients. If the instruction team was not available (e.g. in the weekend) the GP instructed the patient. This information was obtained through interviewing staff

Evaluation at the ED consisted of clinical evaluation plus additional testing according to local hospital protocols; e.g. ABG, chest x-ray, electrocardiogram, blood testing, and D-dimer blood testing if pulmonary embolism was suspected.

#### Oxygen administration

Initiatives differed in how they bridged the time between evaluation of the patient at the ED and arrival of the oxygen concentrator at home. In JB, nurses provided the patients with temporary oxygen cylinders (two litres; available in stock at the ED) until a concentrator was delivered at home. Most patients went home in their private vehicle. The expected delivery time of the concentrator was less than four hours. In AS, the protocol stated that patients had to remain at the ED or hospital ward until the oxygen concentrator was delivered at home. In practice, AS patients would sometimes drive home with temporary oxygen from the hospital, and the concentrator would arrive within four hours similar to the other initiatives. In UT-1 patients were sent home from the ED without temporary oxygen, with arrival of the concentrator also within four hours after arrival at home. In UT-1 type of transportation (per ambulance or private vehicle) from hospital to home was decided on a case-by-case basis. Neither in WH nor UT-2 bridging was needed, as patients were evaluated at home and concentrators were then delivered at home within three and four hours, respectively.

The maximum amount of oxygen administration differed between initiatives (see Table 2). The maximum oxygen treatment in JB was four litres/minute. In WH it was limited to three litres/minute, however if a patient’s oxygen requirement rose above three litres per minute, ED evaluation took place after which patients could return home with increased supplementation if necessary. In AS the limit was three litres/minute (but in consultation with the treating physician this could be higher) and in UT-1 and UT-2 five litres/minute.

#### Instructions of the patient

In all initiatives, patients were clearly instructed about the actions required to safely participate in an acute home-based management program. Those who gave these instructions to the patient varied per site. In JB a nurse and case manager were responsible for instructing the patient, in WH this was the GP, in AS the monitoring team, and in UT-1 and UT-2 a physician-researcher.

### Organization of remote monitoring

#### Monitoring staff

The main differences observed in the design of monitoring are summarized in Table 3. In JB, UT-1 and UT-2, the monitoring centres were staffed by trained medical students (bachelor’s degree minimum). Supervision in JB was done by the patient’s attending GP or the regional GP organisation (during evening night, and weekend). In practice, medical students could also directly approach staff involved in the organization of monitoring for informal clinical questions. In WH, the monitoring team was staffed by nurses supervised by the GP. In AS it consisted of physicians and specialised pulmonology nurses, supervised by a pulmonologist. This monitoring team was authorised to make medical decisions without consultation of the supervisor, while in the four other initiatives medical decisions were only made after consulting the supervisor. In UT-1 and UT-2 the monitoring team was supervised by a GP who was affiliated with the research team. In case of protocol deviation the responsible physician, either the GP (UT-2) or pulmonologist/internist (UT-1), was contacted.

**Table 3:** Organization of the remote monitoring

	JB	WH	AS	UT-1^a^	UT-2^a^
Monitoring staff	Trained medical student^b^	Nurses^b^	Physicians and pulmonary nurse specialist	Trained medical student	Trained medical student
Monitoring centre’s supervisor	GP	GP	Pulmonologist	Study affiliated GP	Study affiliated GP
Opening hours monitoring centre, opening hours, 7 days a week	8 am to 9 pm	9 am to 10 pm	8 am to 9 pm	9 am to 5 pm	9 am to 5 pm
Vital signs included in the monitoring					
Oxygen saturation	Yes				
Temperature	Yes				
Physical condition^c^	Yes				
Respiratory rate	Yes	Yes	Yes	No	No
Heart rate	No	Yes	No	Yes	Yes
SOB score	Yes	Yes	No	Yes	Yes
Coughing score	Yes	No	No	Yes	Yes
Frequency of reporting of vitals (times daily)	3	3	4	3	3
Type and frequency of reviewing vital signs during a day					
Direct review of alerts in app	No (check every 30 min)	Yes	Yes	Yes	Yes
Standard review of app (times daily)	3	0	4	3	3
Standard phone call (number of times daily)	0	0	0	1 if app is used 2 if paper diary is used	1 if app is used 2 if paper diary is used
Home visits (day 0–3)					
Day 0		GP	Nurse	Nurse^d^	Nurse
Day 1	GP	GP^e^	-	Nurse	Nurse
Day 2	GP (phone)	GP^e^	-	Nurse	Nurse
Day 3	GP (phone)	GP^e^	-	-	-
Number of additional monitoring days after discontinuation of oxygen	At the discretion of the GP	2	7	2	2

*Abbreviations: GP* General practitioner, *App* Application, *SOB* Shortness of breath

^a^UT-1 = non-frail patients who would otherwise have been treated in-hospital, UT-2 = frail patient wanting to remain at home

^b^This information was obtained through interviewing staff

^c^As determined by the question: ‘Are you feeling worse than yesterday?’

^d^In practice, the need for a nurse home visit was evaluated on a case-by-case basis

^e^In practice, GPs sometimes decided to follow-up patient by phone

#### Vital signs monitored

Pulse-oximetry, temperature measurement and a general question on well-being (‘do you feel better than yesterday?’ yes/no) were used in all initiatives. Heart rate was routinely registered in WH, UT-1, and UT-2. Respiratory rate was included in the self-measurements in JB, WH, and AS. JB, UT-1, UT-2 included a shortness of breath (SOB) score and coughing score. In WH only SOB score was used, and in AS no subjective scores were applied.

#### Monitoring routine

In all initiatives except WH the vital signs entered by the patient were checked routinely (three to four times daily on set time points) by the monitoring centre. In WH, vital signs were only checked in case of “alerts” (in case a vital sign deviated from the norm). Not all monitoring centres were open in the evening. Notably, in UT-1 and UT-2 patients were instructed to actively call the GP out-of-hours service after the monitoring centre closing time (5 pm). In addition to remote monitoring by the monitoring centre, monitoring home visits were performed by the GP in JB and WH, and by specialised nurses in AS, UT-1 and UT-2.

#### Communication device

All initiatives offered remote monitoring via a smart phone application (Luscii Healthtech BV) [15]. Two initiatives (UT-1 and UT-2) offered an additional paper diary option for patients who preferred follow-up via telephone, e.g. due to absence of digital literacy.

#### Stop criteria for monitoring at home

All initiatives except JB defined stop criteria for monitoring at home. In WH, UT-1 and UT-2 home monitoring could be terminated if patients had SpO2 ≥ 94% without supplementary oxygen for at least 48 hours. In AS, patients were discharged from home monitoring if they had SpO2 > 94% without oxygen for at least seven days (Table 3).

### Organization of the nursing care at home

In UT-1 and UT-2 a nurse visit was scheduled on day 0, 1 and 2 of home management, for clinical assessment, verification of medication adherence and verification of adequate equipment use. In AS, a nurse specialist visited the patients once to check the understanding of the protocol, to answer any questions, and to perform a glucose check. In both JB and WH nursing care was not standardised but could be arranged if needed (e.g. in case of patient or caregiver insecurity) or was continued if a patient already received home care outside of the initiative.

### Devices used

All patients received oxygen treatment via a nasal cannula and an oxygen concentrator. Delivery of this equipment was outsourced to various nationally available suppliers. In UT-1 and UT-2 the specific brand of concentrator was protocolised (DeVilbiss Healthcare LLC). JB, WH and AS did not standardise the concentrator brand. In WH, the choice of concentrator depended on availability; due to high demand and shortage, supplied brands could differ. A summary of the used medical devices can be found in Table 4.

**Table 4:** Medical devices in the home-based initiatives

	JB	WH	AS	UT-1^a^	UT-2^a^
Pulse-oximeter Brand	MediSana PM100	HUM AEROcheck	Westfalen Hb0 Smart	Nonin type 3230 or iHealth air pulse	Nonin type 3230 or iHealth air pulse
Thermometer	Purchased by the patient [preferably ear or rectal]	Purchased by the patient [preferably ear]	Purchased by the patient [preferably ear or rectal]	Braun Thermoscan 3 [ear]	Braun Thermoscan 3 [ear]

^a^UT-1 = non-frail patients who would otherwise have been treated in-hospital. UT-2 = frail patient wanting to remain at home

### Workload of the general practitioner

The patients’ GPs were assigned different roles and subsequently experienced different workloads in the various home-based management initiatives. The burden on the GP was highest in JB, WH and UT-2, where GPs were medically responsible and, in JB and WH, also supervised monitoring centre’s staff (Supplementary Table 4). In JB patients could only be treated at home if their GP was willing to clinically evaluate the patient the day after the start of home management, also if the day after was a Saturday. In WH the GP was the one who organized the oxygen treatment at home, whereas in other initiatives this was organized by the hospital or an external coordination centre, in order not to burden the GP with organizational activities.

## Discussion

### Main results

We identified and systematically described five Dutch acute home management initiatives for hypoxemic COVID-19 patients. All initiatives provided pulse-oximetry and an app for transferring vital signs for review by a monitoring centre. Three initiatives included low-risk patients and two were designed specifically for frail patients. There were differences in how the interprofessional collaboration in the initiatives was organized.

### Relation to previous studies

Recent studies have evaluated ‘early discharge’ with home management of COVID-19 patients [8, 11, 16, 17] or home monitoring without oxygen treatment [17–19].

In a descriptive, cross-sectional study outlining the primary health care pathway for COVID-19 in 30 European countries, it was mentioned that six out of 30 countries prescribed oxygen therapy in primary care, presumably at home. These data were acquired through questionnaires, and details about the organization of care are lacking [20]. A retrospective study by Serafini et al. (2023) was aimed at evaluating the COVID-19 management strategies of Italian GPs. Out of 5340 patients from 46 GPs, 202 (4%) received oxygen therapy as treatment, more than 85% of patients with severe or critical disease were remotely monitored, and 52% received a home evaluation. Although the insight into the design decisions and organizational structures is limited, this study indicated that home management of acutely ill patients was successfully put into practice [21].

Several studies specifically described acute home management initiatives with a protocol for oxygen treatment and remote monitoring, however, some of them did not report whether patients actually received supplemental oxygen [22, 23]. Sitammagari et al. (2021) described a virtual acute care unit in the United States designed to provide oxygen treatment and hospital-level care interventions. A total of 41 of the 184 patients treated in this virtual acute care unit required oxygen treatment at home [10]. In Pakistan a study performed by Alishan et al. (2022) reported outcome data on healthcare workers infected with COVID-19 that were given home monitoring and treatment. Five of the 128 treated patients required oxygen treatment; 15 patients were hospitalised [24]. These studies indicate that acute home treatment with oxygen and remote monitoring is being developed worldwide, however, none of these studies provided sufficient and detailed information on the organizational structure of the home management such as the eligibility of the included patients, organization of monitoring, nursing care or devices used. Comparing our results with these studies in this respect is therefore not possible. Banjeree et al. (2021) included patients in the United States with COVID-19 pneumonia either discharged from the ED or from “inpatient encounters” who were treated with low amounts of oxygen at home [25]. Approximately 25% of patients received acute home treatment (75% were discharged early from the ward). The authors describe that the hospital stocked oxygen concentrators and pulse-oximeters for patients to take home directly from the ED after office hours. None of the described Dutch initiatives had this option, but it could be a good option for bridging the time between ED discharge and arrival at home after hours. The authors also mention that nurses provided continuous telephone follow-up with physician back-up; comparable to most of the Dutch initiatives.

Schiff et al. (2022) describe an initiative in the United Kingdom for frail patients with COVID-19 [26]. The medical treatment possibilities included intravenous fluids, dexamethasone, thrombosis prophylaxis and oxygen treatment (it is not reported how many patients received oxygen, likely the patients described to have “severe COVID-19” [*n* = 42]). Initially they delivered oxygen treatment through a centralised ordering system but the 4 hour wait appeared to be too long; on one occasion an ambulance had to wait with the patient to provide temporary oxygen. The team then started using short-term portable cylinders which they had stocked in their “urgent response” car to provide direct oxygen treatment if necessary. The authors mention that analogous to the in-hospital setting, patients received a minimum of one medical review daily with treatment plan revision. This is possibly more stringent than in most Dutch initiatives, where only in the WH protocol a daily physical review was described. In other initiatives the patient was reviewed daily by phone or by a nurse visit, or only reviewed the first day of home manangement.

### Strength and limitations

To our knowledge this is the first overview of acute COVID-19 home-based management programs. We have provided detailed descriptions in which we highlight divers organizational and management choices of home management to use as examples.

Several limitations of our study need to be discussed. First, we may have missed a program because we remained unaware of it despite our call for protocols through the Dutch General Practice Research Consortium. Second, given the dynamic of the COVID-19 pandemic, the reported protocols may have been adapted overtime. To improve the validity of our overview we contacted all healthcare centres’ staff members involved in the development of the described five initiatives to obtain additional information. Authors from all initiatives checked the collected data and participated in the final overview.

### Implications for practice

Our overview and assessment can help healthcare organizations when setting up or implementing acute home management initiatives, for COVID-19 or other acute diseases in the near future.

#### Patient selection, instruction and monitoring

Acute home-based management programs can be focused on different patient categories. It may benefit both frail patients that prefer to not be hospitalised, as well as patients who are expected to have an uncomplicated disease course and would otherwise be hospitalised. In the first patient category, patients profit from remaining in the their own environment. Their attending GP profits from the program because of the support from the remote monitoring centre. Acute home management of the latter category of patients also has the potential to reduce hospitalisation rates resulting in cost benefits.

All initiatives recommended a maximum of 3 to 5 L oxygen per minute for both categories of patients and organized onsite instruction for patients at the start of treatment. Instruction could be given by a dedicated instruction team, a nurse at the ED, a case manager, or a monitoring team member. All initiatives verified the patient’s understanding of the information at a protocolled time during a nurse of GP home visit, either the same day or the day after start of home management. This is a safety netting strategy that seems advisable in such an acute care setting.

The optimal number of routine monitoring contacts remains to be determined. Acute home management with remote monitoring is less labour-intensive for health care providers than hospital admission, and more people can be managed. A disadvantage is that medical deterioration can more easily remain undetected or be discovered too late, notably if monitoring values remain within the norm or when devices perform (or are used) inadequately. Future studies should focus on the impact of organizing more or fewer contact moments on safety and patient satisfaction.

#### Workload for professionals

GP workload is mainly determined by the burden of organizing the (oxygen) treatment and monitoring. In one initiative the GP arranged the delivery of the oxygen concentrator at home, whereas in other initiatives this was organized by the hospital or an external coordinating centre. In three initiatives, the GP was the medically responsible physician, as opposed to a hospital specialist. In two of these initiatives this was the only possible option since patients did not wish to go to the hospital. In JB, however, patients did go to the ED for in hospital evaluation, and medical responsibility was then transferred back to the GP when the patient was in home management (substitution of care) because the GP was likely more familiar to the patient, and physically closest. In AS, on the contrary, the specialist remained the responsible physician for patients at home (extramural hospital care).

All Dutch remote monitoring centres were open 7 days a week. In case of early closure of the centre (5 pm), the workload for GPs on call at out-of-hours service may have been somewhat higher than usual.

#### Protocol versus practice

Our overview describes “work as imagined” and not necessarily of “work as done” [27]. Certain items of the protocols will not accurately reflect real-life practice. E.g. in UT-1 and UT-2 there were (i) difficulties delivering oxygen concentrators on time, (ii) challenges with availability of home nurses and (iii) occasional miscommunication between the treating physicians and the out-of-hours GP services. In JB, WH, AS the oxygen concentrators were generally delivered on time. Interestingly, in the region of WH there was ample availability of nursing care (JB and AS initiatives did not involve protocolised home care nurses). In WH miscommunication between the treating physicians and the out-of-hours GP services was also an issue, in JB communication out-of-hours GP services was generally satisfactory (in AS the out-of-hours GP service was not involved).

### Future research

Acute home management can be considered as a complex intervention [28], with involvement of multiple settings, professionals and organizations. Differences between initiatives should be interpreted as being context-related. Future research aimed at understanding which context factors are preconditional for setting up well functioning acute home management initiatives will spur further development of acute home management initiatives. Yet, the first next step needed is to perform a prospective, multi-context outcome study to determine how the different organizational and management choices impact patient outcomes, patient satisfaction, and healthcare costs.

## Conclusions

There are differences in the organization of interprofessional collaboration for acute home management of hypoxemic COVID-19 patients, but all initiatives used pulse-oximetry and an app for remote monitoring. Our overview provides healthcare providers and organizations with help setting up and implementing similar acute home management initiatives for COVID-19 or other diseases with a critical episode that would otherwise need hospital care.

### Supplementary Information

**Additional file 1: Supplementary Table 1.** Motivation for the exclusion of initiatives. **Supplementary Table 2.** Temporal context of included initiatives. **Supplementary Table 3.** Medical care details. **Supplementary Table 4.** Overview of the GP’s role and workload.

## Data Availability

The protocols used and analysed during the current study are available from the corresponding author on reasonable request.

## Abbreviations

ABG: Arterial blood gas
AS: Albert Schweitzer Hospital Dordrecht
App: Application
BMI: Body Mass Index
COPD: Chronic Obstructive Pulmonary Disease
ED: Emergency department
GP: General practitioner
JB: Jeroen Bosch Hospital Den Bosch
SpO2: Peripheral capilary oxygen saturation
SOB: Shortness of breath
UT-1: Utrecht (protocol 1)
UT-2: Utrecht (protocol 2)
WH: Wilhelmina Hospital Assen
