# Supplementary material for: Home-based initiatives for acute management of COVID-19 patients needing oxygen: differences across The Netherlands
Source: BMC Health Serv Res. 2023 Nov 15;23:1257. doi: 10.1186/s12913-023-10191-6 (PMC10652550; doi:10.1186/s12913-023-10191-6)
Supplement: Supplementary file 1 — Additional file 1: Supplementary Table 1. Motivation for the exclusion of initiatives. Supplementary Table 2. Temporal context of included initiatives. Supplementary Table 3. Medical care details. Supplementary Table 4. Overview of the GP’s role and workload. [file 12913_2023_10191_MOESM1_ESM.docx]

Supplementary table 1: Motivation for the exclusion of initiatives.

| Initiating centre (region) | Reason for exclusion |
| --- | --- |
| General practitioner cooperation Westelijke Mijnstreek (Limburg) | No patients were treated according to this treatment plan |
| Leiden University Medical Center (Leiden) | Patients did not receive oxygen treatment at home, only remote monitoring. |
| Radboud Medical Center and Canisius Wilhelmina Hospital (Nijmegen) | Remote monitoring was not part of the protocol. |
| Isala Hospital (Zwolle) | Remote monitoring at home and treatment with oxygen were separate initiatives that were not connected. |

Supplementary table 2: Temporal context of included initiatives.

|  | JB | WH | AS | UT-1 | UT-2 |
| --- | --- | --- | --- | --- | --- |
| Initiative runtime^1^ | February 2021 – June 2022 | December 2021 – April 2022 | December 2020 – February 2022 | November 2021 – March 2023 | November 2021 – March 2023 |
| Ongoing implementation | Yes* | No | No | No | Yes** |
| Major revisions of protocol during implementation | No | No | No | No | No |

*^1^The WHO declared COVID-19 a global pandemic in March 2020, with three consecutive pandemic waves in the Netherlands: (1) March 2020 through May 2020; (2) October 2020 through January 2021; (3) February 2021 through June 2021.*

**The program may be integrated in a home monitoring/management program for other (infectious) disease in 2023-2024.*

***After the initial research/pilot phase, home management was further implemented into usual care.*

Supplementary table 3: Medical care details.

| Medical guideline content | JB | WH | AS | UT-1^1^ | UT-2^1^ |
| --- | --- | --- | --- | --- | --- |
| Dexamethasone  Dose: 6 mg / day; 10 days | Yes | Yes | Yes | Yes | Yes |
| Thrombosis prophylaxis  [LMWH dose in IU / day] | Fraxiparin  [5700] | Dalteparin  [5000] | Dalteparin  [5000] | Dalteparin  [5000] | Dalteparin  [5000] |
| Proton Pump inhibitor | Yes  20 mg / day | No | Yes | Yes | Yes |
| Glucose checks | Yes | If considered necessary by GP | Yes | Yes^2^ | Yes^2^ |
| Antibiotics | No | Recommended if CRP > 100 mg/L | No | No^3^ | No^3^ |

*Abbreviations: LMWH = Low Molecular Weight Heparin, IU=international units, GP = general practitioner, CRP = C-reactive protein.*

*^1^UT-1 = non-frail patients who would otherwise have been treated in-hospital, UT-2 = frail patient wanting to remain at home.*

^2^Protocol was amended after a trial period; glucose checks were only recommended in case of pre-existing diabetes hereafter.

^3^Only recommended in case if strong suspicion of bacterial superinfection.

Supplementary table 4: overview of the GP’s role and workload.

|  | | JB | WH | AS | UT-1^1^ | UT-2^1^ |
| --- | --- | --- | --- | --- | --- | --- |
| GP responsible for eligibility screening of patients | | Yes | Yes | No | Yes | Yes |
| GP is the medically responsible and supervises monitoring centre’s staff | | Yes | Yes | No | No | Yes |
| Protocolized patient contacts by GP | |  | | | | |
|  | Home visits | Day 1 | Daily^2^ | - | - | - |
|  | By phone | Daily thereafter | - | - | - | - |
| Review of vital signs by GP | |  | | | | |
|  | Daily with app | Yes | Yes | No | No | No |
| Organization of logistics by GP | |  |  |  |  |  |
|  | GP responsible for providing patient’s information to the general out-of-hours GP centre | Yes | Yes | N/A^3^ | Yes | Yes |
|  | O2 delivery | No | Yes | No | No | No |

*Abbreviations: GP = general practitioner, App = application, N/A = not applicable.*

*^1^UT-2= frail patient wanting to remain at home, UT-1= non-frail patients who would otherwise have been treated in-hospital.*

*^2^In practice, GPs sometimes decided to follow-up patient by phone*

*^3^Patients were instructed to call the hospital directly in case of questions, instead of the out-of-hours GP centre.*
